# Case report: whole genome sequencing based investigation of maternal-neonatal listeriosis in Sichuan, China

**DOI:** 10.1186/s12879-019-4551-9

**Published:** 2019-10-26

**Authors:** Lijuan Luo, Xi Chen, Michael Payne, Xiaolong Cao, Yan Wang, Jie Zhang, Jianping Deng, Hong Wang, Zhengdong Zhang, Qun Li, Ruiting Lan, Changyun Ye

**Affiliations:** 1 State Key Laboratory of Infectious Disease Prevention and Control, National Institute for Communicable Disease Control and Prevention, Collaborative Innovation Center for Diagnosis and Treatment of Infectious Diseases, Chinese Center for Disease Control and Prevention, Beijing, 102206 China; 2 Zigong Center for Disease Control and Prevention, Zigong, 643000 China; 3 School of Biotechnology and Biomolecular Sciences, University of New South Wales, Sydney, NSW 2052 Australia; 4 Beijing Changping Institute for Tuberculosis Prevention and Treatment, Beijing, 102206 China

**Keywords:** Case report, Maternal-neonatal listeriosis, Whole genome sequencing, Plasmid

## Abstract

**Background:**

Neonatal listeriosis is a rare but severe disease manifesting as septicemia and central nervous system (CNS) infections with a high fatality rate of around 20 to 30%. Whole genome sequencing (WGS) is a promising technique for pathogen identification and infection source tracing with its high resolution.

**Case presentation:**

A case of neonatal sepsis with listeriosis was reported with positive blood culture for *Listeria monocytogenes*. The case was investigated to confirm the vertical transmission of the infection and identify the potential food source of the maternal *L. monocytogenes* infection using WGS. *L. monocytogenes* was isolated from the neonate’s blood sample the day after caesarean delivery and from the mother’s genital and pudenda swab samples 5 days and 13 days after caesarean delivery. WGS showed that the isolate from the neonate was identical to the genome type of the isolates from the mother, with only one of the 4 isolates from the mother differing by one single nucleotide polymorphism (SNP). By WGS, one *L. monocytogenes* isolate from a ready-to-eat (RTE) meat sample in the patients’ community market shared the same sequence type but was ruled out as the cause of infection, with 57 SNP differences to the strain causing the maternal-neonatal infection. The food isolate also carried a novel plasmid pLM1686 that harbored heavy metal resistance genes. After caesarean section, the mother was treated with a third generation cephalosporin which *L. monocytogenes* is naturally resistant to, which may explain why genital and pudenda swabs were still culture-positive for *L. monocytogenes* 13 days after delivery.

**Conclusions:**

Genital swab culture for *L. monocytogenes* had been informative in the diagnosis of maternal listeriosis in this case. The high resolution of WGS confirmed the maternal-neonatal transmission of *L. monocytogenes* infection and ruled out the *L. monocytogenes* contaminated RTE meat from the local market as the direct source of the mother’s infection.

## Background

Neonatal listeriosis is an infection caused by *Listeria monocytogenes*, leading to sepsis and meningitis with a high fatality rate around 20 to 30% [1, 2]. Early-onset neonatal listeriosis (defined as infection in neonates with less than 7 days of age) is mostly caused by vertical transmission from the mother [3, 4]. *L. monocytogenes* can traverse the placenta and infect the fetus, resulting in abortion, stillbirth, premature birth and neonatal listeriosis [5]. However, maternal listeriosis is relatively mild in severity manifesting as flu-like symptoms (such as fever, myalgia, nausea and vomiting) with 30% of maternal listeriosis patients being asymptomatic [1]. Thus, it is difficult to recognize maternal-neonatal listeriosis due to the mild or asymptomatic maternal listeriosis before it develops into life threatening conditions of neonatal bacteremia or meningitis through vertical transmission. While neonatal sepsis and meningitis cases caused by *L. monocytogenes* are relatively rare (2 to 7% of infections) [6, 7], it is vital to increase clinicians’ awareness on maternal-neonatal listeriosis for early diagnosis and effective targeted treatment, especially in countries such as China where listeriosis is less recognized and there are no surveillance systems in place [8].

*L. monocytogenes* is a common foodborne pathogen [9]. Outbreaks may be traced back to the food source and whole genome sequencing (WGS) has been demonstrated as a powerful tool for source tracing of *L. monocytogenes* outbreaks [10]. In this study, we investigated a case of maternal-neonatal listeriosis using WGS to confirm vertical transmission and to identify the potential food source of the maternal *L. monocytogenes* infection.

## Case presentation

**Fig. 1:**
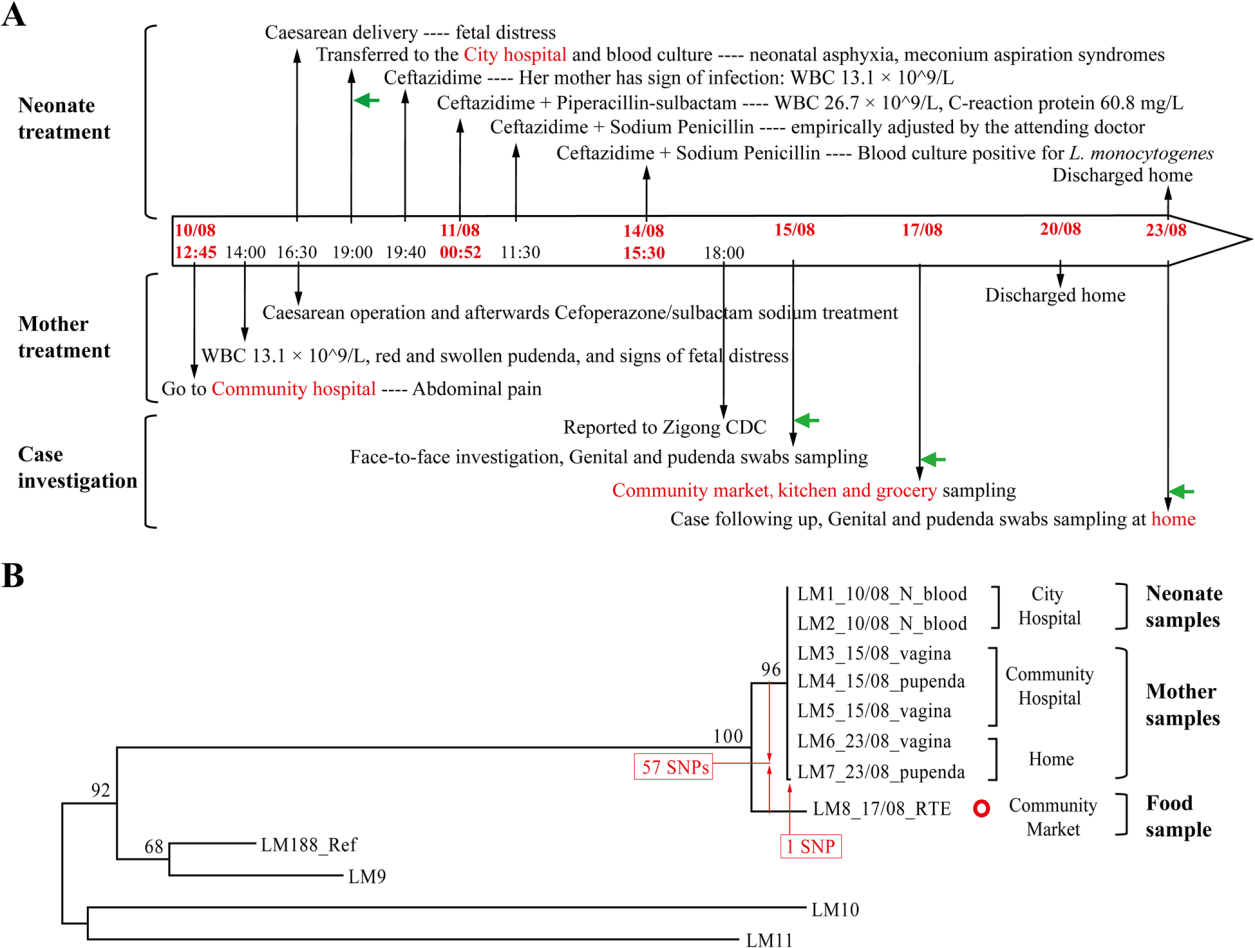
The fetal-maternal listeriosis case diagnosis, treatment, investigation and WGS analysis. **a** Timeline of the maternal-neonatal listeriosis diagnosis, treatment and investigation since 10/08/2015. The diagnosis and treatment of the neonate and the mother, as well as the process of investigation are indicated in the upper, middle and lower sections of the timeline, respectively. The time points of sampling for *L. monocytogenes* culture are marked with arrows. Treatment and sampling locations are highlighted in red. **b** The phylogenetic analysis of the *L. monocytogenes* strains from the patient and the food products. The maximum parsimony tree was constructed with the software Mega 5.04, with 1000 bootstrap replicates. With the sequence of LM188 as the reference, the neonate-blood-source isolates (LM1 and LM2_N_blood) which were cultured on 11/08/2015, were identical with the isolates obtained from the vagina and pudenda of the mother. There was one SNP mutation in the strain (LM7) isolated from the pudenda of the mother after she had been discharged. The RTE-meat-source strain (LM8) isolated from the community market had 57 SNP differences compared to the neonate infection strain (LM1). The food source strain LM8 harbors a novel plasmid named pLM1688 (marked as a ring)

A 21-year-old pregnant woman without a significant medical history, was admitted to the local community hospital with lower abdominal pain at 36 weeks gestation in Sichuan, China. Her axillary temperature was 37.7 °C. Physical examination found that her labium majus pudendi was red and swollen, with moderate pain. White blood cell (WBC) count was 13.1 × 10^9^/L. A female afebrile baby was born prematurely by cesarean section because of intrauterine distress. After the cesarean, the mother was treated intravenously with cefoperazone/sulbactam sodium 3.0 g in 100 mL saline twice a day and was discharged from hospital after 10 days (Fig. 1a). The neonate showed signs of asphyxia and meconium aspiration syndrome after birth and was transferred to a higher tier city hospital. Considering that the mother presented with signs of infection prior to delivery, the neonate’s WBC count was 26.7 × 10^9^/L and C-reactive protein was 60.8 mg/L, neonatal sepsis was strongly suspected. The neonate was empirically treated with sodium penicillin of 260,000 units (around 100,000 units/kg/dose) and ceftazidime 134 mg (50 mg/kg/dose), both of which were administered intravenously every 12 h. Blood culture was performed when the neonate entered the hospital and *L. monocytogenes* was isolated and identified 4 days later. Lumbar puncture and cerebrospinal fluid culture were not performed, therefore neonatal meningitis cannot be excluded. After 13 days’ treatment, the neonate’s C-reactive protein level returned normal and blood culture was negative for *L. monocytogenes*. The neonate was then discharged from the hospital (Fig. 1a).

This listeriosis case was reported to the local Center for Disease Control and Prevention, and the following investigations were carried out. Blood and milk samples, vaginal fornix and pudenda swabs from the mother were collected and cultured for *L. monocytogenes* in the community hospital (Fig. 1a). *L. monocytogenes* was isolated from the vaginal fornix and pudenda swabs while blood culture and breast milk culture were negative. Culture for *L. monocytogenes* from stool samples from the mother was not performed. The patient, who had been discharged home, was followed-up at home 13 days after the delivery. Vaginal fornix and pudenda swabs were again collected from the mother and were positive for *L. monocytogenes*. An extensive source tracing of the *L. monocytogenes* infection was performed. A face-to-face interview was conducted to determine the mother’s food exposure history during the previous 1 month prior to the premature delivery. The mother had a history of consuming ice cream, ready-to-eat (RTE) meat and salads. Food and environmental samples from her home (30 samples including the fridge food and fridge surface swabs, kitchen and bathroom environmental swabs), RTE meat, RTE salads, raw pork, beef and frozen chicken samples from the patient’s community market (52 samples), and ice cream in the local grocery store (3 samples) were collected (Fig. 1a). One RTE meat sample and one frozen chicken sample from the community market were positive for *L. monocytogenes* while all other samples were negative.

All clinical isolates (2 neonate isolates and 5 mother isolates) and two food isolates of *L. monocytogenes* were subjected to multi-locus sequence typing (MLST) [11]. All human isolates and one RTE meat isolate belonged to sequence type 87 (ST87), one raw chicken isolate belonged to ST9. The chicken isolate was not closely related to the human cases as it belonged to a different ST while the RTE meat isolate had the same ST and may be related which required further assessment using a higher resolution method.

In order to confirm the vertical transmission of listeriosis and resolve the relationship between the RTE food isolate and the patient isolates, the genomes of all 8 ST87 isolates were sequenced by Illumina sequencing. Using the complete genome ICDC-LM188 (GenBank accession No. CP015593.1) as the reference, SNPs were called using Burrows-Wheeler Aligner (BWA-MEM) method [12]. At least 10 reads and a coverage of 70% were required to call a SNP. All human isolates from the neonate and the mother were identical except for one isolate from the mother (LM7). LM7, isolated from the second pudenda swab (13 days after the caesarean), differed from the other clinical isolates by a single SNP (Fig. 1B). However, the RTE meat *L. monocytogenes* isolate showed 57 SNP differences (56 single base mutations and one single base deletion) from the main genome type of the clinical isolates. Among the 57 SNP differences, there were 11 located in the core genome of *L. monocytogenes* (Additional file 1: Table S1)*.* Considering the mutation rate of 0.4 SNPs per core genome per year [13], the human isolates would have been separated from the food isolate around 27.5 years ago (Fig. 1b). By core-genome MLST (cgMLST) analysis, *L. monocytogenes* isolates from the baby and the mother (LM1 to LM7) all belonged to the same cgMLST type: L1-SL87-ST87-CT5541. Note that LM7 from the mother has one allele difference to the other isolates (LM1 to LM6) reflecting the single SNP difference observed, but both were assigned the cgMLST type as *L. monocytogenes* strains with no more than 7 alleles are considered to be epidemiologically linked and are assigned to the same cgMLST type [13], although this cutoff is debatable. The RTE meat strain belonged to L1-SL87-ST87-CT5542 (Additional file 1: Table S2). Therefore, the *L. monocytogenes* contaminated RTE meat from local market was not the source of the mother’s infection.

Analysis of virulence genes showed that the isolates from the patients and RTE food, all of which belonged to ST87, harbored the newly discovered *Listeria* pathogenicity island 4 (LIPI-4) [14], and intact *InlA* and *inlB* genes that encode the invasive proteins internalin A and internalin B respectively [15] were present.

**Fig. 2:**
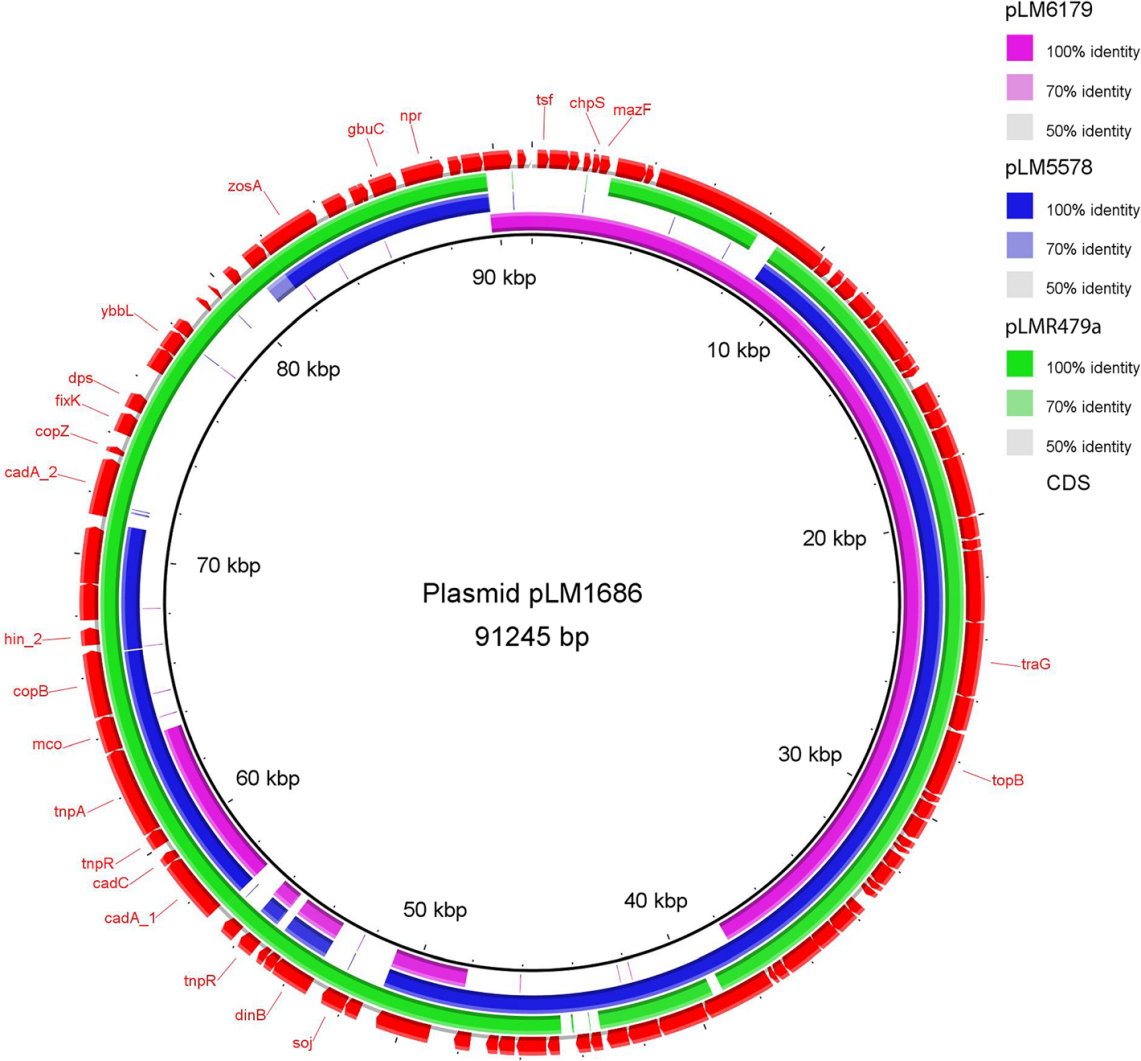
The plasmid comparison using BRIG with the plasmid pLM1686 as the reference. The newly found pLM1686 (being set as the reference) was compared to previously described plasmids pLM1679, pLMR5578 and pLM479a, represented in purple, blue and green, respectively. The outer red ring refers to the annotation of pLM1686 with the known genes indicated

Additionally, the RTE meat isolate (LM8) carried a novel plasmid, named pLM1686. By assembling the raw Illumina reads of LM8 using SKESA v2.3, we identified pLM1686 as a circular plasmid [16]. The plasmid was annotated using Prokka v1.12, and compared with reported plasmids of *L. monocytogenes* using Roary v3.11.2 with an identity of 70% at nucleotide level as cut-off [17, 18]. Plasmid pLM1686 was found to be most similar to the previously reported *Listeria* plasmid pLMR479a [19]. Among the 91 annotated genes of pLM1686, 79 genes were present in pLMR479a, including the heavy metal resistance genes (Fig. 2) (Additional file 1: Table S3). The remaining 12 genes of pLM1686 that were absent in pLMR479a, were present in either plasmid pLM6179 or pLM5578 (Fig. 2) (Additional file 1: Table S3) [20, 21].

## Discussion and conclusions

Maternal-fetal listeriosis is a rare but severe infection [4]. *L. monocytogenes* is one of the few foodborne pathogens that can be vertically transmitted to the unborn fetus [2]. In this case, WGS confirmed the vertical transmission with the neonatal isolate being identical to maternal isolates. *L. monocytogenes* is thought to reach the placenta most commonly via hematogenous spread from the gastrointestinal tract to the placenta [5]. However, the exact route of transmission for this case was uncertain. Blood culture from the mother was negative for *L. monocytogenes* and the placenta was not sampled. Therefore, the hematogenous route cannot be confirmed. On the other hand, vaginal fornix swabs and pudenda swabs were positive. It is possible that *L. monocytogenes* may have reached the fetus via an ascending route from the genital tract, which has rarely been reported [22], resulting in fetal infection and distress. The infected fetus displayed signs of distress, which increases the risk for passage of meconium, aspiration and neonatal asphyxia. As such, the infant showed evidence of meconium aspiration syndrome and neonatal asphyxia after birth, indicating a dangerous course of infection.

Diagnosis of maternal and neonatal listeriosis as early as possible is critical to improve prognosis [23]. Maternal listeriosis cases may only show mild symptoms with 30% cases being asymptomatic [1]. Therefore, maternal listeriosis cases can easily be overlooked until the fetus has developed listeriosis or the mother presents with obstetric symptoms. In this case, the pregnant woman showed symptoms of colpitis, and vaginal/pudenda swabs were culture-positive for *L. monocytogenes*. This highlights that for suspected maternal-fetal listeriosis cases, non-invasive cervical/genital swabs culture for *L. monocytogenes* may assist in diagnosis of fetal listeriosis, in addition to maternal blood culture [5, 15]. A recent study reported that 55% of maternal blood samples and 26% of maternal cervical or vaginal swabs in maternal-neonatal listeriosis cases were culture positive for *L. monocytogenes* [4]. Placenta samples and gastric aspirates from neonates showed the highest rate of positive culture for *L. monocytogenes* in maternal-fetal listeriosis cases [4]. Therefore, during the delivery, placental tissue for *L. monocytogenes* culture, would be most valuable in assessing whether neonatal listeriosis resulted from vertical transmission.

The recommended first line empirical therapy for suspected early onset neonatal sepsis in many countries is ampicillin plus gentamicin which cover *L. monocytogenes* [24, 25], while in China penicillin combined with a third-generation cephalosporin are commonly used [26]. For the empirical therapy of suspected meningitis, ampicillin plus third-generation cephalosporin are recommended, and cerebrospinal fluid culture is required to guide appropriate therapy [25]. In this case, the neonatal sepsis was empirically treated with penicillin G and ceftazidime, which was appropriate because neonatal meningitis was suspected. It should be noted the patient’s family objected to lumbar puncture for cerebrospinal fluid analysis on the baby and meningitis was not confirmed. Since *L. monocytogenes* is sensitive to penicillin G but naturally resistant to cephalosporins [2], ceftazidime should have been removed from the neonate’s treatment regimen after *L. monocytogenes* infection was confirmed from blood culture.

Treatment for maternal listeriosis is mostly focused on the antepartum treatment of pregnant women to prevent fetal/neonatal listeriosis [2, 27]. Postpartum treatment for mothers after delivery has rarely been discussed in the literature, as the mother can recover without treatment [2]. In this case, the mother was given cefoperazone/sulbactam after the cesarean which was inappropriate treatment for listeriosis, as *L. monocytogenes* is naturally resistant to cefoperazone (a cephalosporin) and penicillin is the primary antibiotic for the treatment of listeriosis [2, 28]. Although the patient made an uneventful recovery, it was noteworthy that the vaginal fornix and pudenda swabs were still positive for *L. monocytogenes* 13 days after delivery. It has been reported that among the mothers that didn’t receive appropriate antibiotic therapy for listeriosis (although only 10 cases), more than half of their infants developed late onset disease [4]. This case highlights why more attention should be paid to the appropriate postpartum treatment of maternal listeriosis.

The mother was most likely initially infected by a single strain. Among the isolates from the mother and the neonate, there was only one SNP mutation observed in the pudenda-swab isolate which was isolated 13 days after the cesarean delivery. This mutation may have been present in the earlier period but not sampled or it could also have appeared within the 13-day period. Based on the mutation rate of *L. monocytogenes* (2.5 × 10^− 7^ substitution per site per year) [13], a mutation may arise in as little as 30 days. The single SNP mutation in the core genome may have occurred during the infection period of the mother*.* But it is also possible that the mother initially infected by two related strains of *L. monocytogenes.*

In an attempt to trace the source of infection, food and environment samples from the patient’s home and local market were sampled. One RTE meat *L. monocytogenes* isolate obtained from the patient’s community market was found to have the same ST as the patients’ isolates. However, by WGS analysis, the RTE meat isolate differed from the clinical isolates with 57 SNP differences and belonged to a different cgMLST type from the case, which would have diverged approximately 27.5 years ago. Thus, the RTE food is not a direct infection source of this case. This is not surprising as ST87 is prevalent in China and considerable genomic diversity must have developed [9]. Nevertheless, many reported listeria outbreaks were caused by RTE food, which were consumed without heating [29]. From 2017 to 2018, there was a large scale listeriosis outbreak of 1060 cases in Africa caused by one brand of RTE meat with a mortality rate of 27% [30]. In Canada, two historical outbreaks were confirmed to be caused by RTE crabmeat through WGS analysis [31]. From 2015 to 2016 in Italy, there was one listeriosis outbreak due to *L. monocytogenes* contaminated processed pork products [32]. During 2015 to 2017, one cross-border listeriosis outbreak occurred in Denmark and France was caused by cold-smoked salmon from a third European Union country [33]. In our case investigation, the presence of *L. monocytogenes* in the RTE meat in the patient’s community market was a health risk to the local consumers. Our study highlighted that case investigation can help identify *L. monocytogenes* contaminated food products and prevent further infections.

This WGS-based analysis showcased the power for tracing the infection source, due to its high discriminatory power. This study applied two major genomic tools (SNP analysis and cgMLST typing) for the WGS analysis of the *L. monocytogenes* isolates [12, 13]. The results of both methods were concordant with each other. The standardized nomenclature of cgMLST enables comparison between different studies [13].

Interestingly, a novel plasmid pLM1686 was found in the *L. monocytogenes* isolate from the RTE meat sample while the human isolates did not harbor any plasmids. Plasmid pLM1686 was most closely related to plasmid pLMR479a. Both plasmids harbored heavy metal resistance genes (e.g. *cadA1*, *cadA2*, *cadC* and *copB*) and other genes that are related with environmental adaption [34, 35]. Plasmid pLMR479a is common in the globally prevalent ST8 strains of *L. monocytogenes* [36, 37]. A recent study has confirmed that pLMR479a contributed to stress tolerance of *L. monocytogenes* against disinfectants, acidity, salinity and oxidation [38]. ST87 is a common type of *L. monocytogenes* in either clinic infections or food contaminations in Asia [39–41]. Plasmid pLM1686 may enhance the environmental survival of ST87 *L. monocytogenes* and further studies are required.

Maternal-neonatal listeriosis is a rare but severe vertical transmissible disease caused by the foodborne pathogen *L. monocytogenes.* Genital swab culture for *L. monocytogenes* had been informative in the diagnosis of maternal listeriosis in this case although blood culture was negative. Placenta tissue or placental smear culture should be performed for premature delivery with no known cause and can be of assistance in confirming *Listeria* infection and route of transmission. As *L. monocytogenes* is generally resistant to third generation cephalosporins, they should not be used for treating maternal-neonatal listeriosis. WGS showed high resolution source tracing and confirmation of maternal neonatal transmission of *L. monocytogenes* infection.

## Supplementary information

**Additional file 1: Table S1.** Location of the 57 SNPs between the patient’s isolate and the RTE food isolate. The SNP differences and the location of the 57 SNPs between the RTE food isolate (LM8) and the patient’s isolate (LM1) were listed using LM188 (GenBank accession No. CP015593.1) as the reference. The SNPs on the core genes of *L. monocytogenes* were also indicated. **Table S2.** The cgMLST types of the patients and food source *L. monocytogenes* strains isolated at different time point in this case investigation. The cgMLST types of the patients and food source *L. monocytogenes* were assigned by the cgMLST database (https://bigsdb.pasteur.fr), with allele profiles offered) **Table S3.** The annotation of plasmid pLM1686 and the distribution of the genes in the other three online plasmids (identity 70%). The circular sequence of the plasmid pLM1686 was assembled with SKESA v2.3 using the raw reads of Illumina sequencing. The plasmid sequence was annotated with Prokka v1.12. The annotation of the pLM1686 were compared with the online plasmids of *L. monocytogenes* using Roary v3.11.2 with the identity of 70% as the cut-off. Plasmid pLM1686 was found to be most similar to the previously reported *Listeria* plasmid pLMR479a sharing 79 genes. The remaining 12 unshared genes of pLM1686 with pLMR479a, were present in either plasmid pLM6179 or pLM5578.

## Data Availability

The genome assembly sequences of the strains and the plasmid pLM1686 were deposited at GenBank under the Bioproject of PRJNA503772 and PRJNA447903. All data generated or analysed during this study are included in this published article and its supplementary information files.

## Abbreviations

cgMLST: core genome MLST
CNS: Central nervous system
LIPI-4: *Listeria* pathogenicity island 4
LM: *Listeria monocytogenes*
MLST: Multilocus sequence typing
pLM: Plasmid of *Listeria monocytogenes*
RTE: Ready-to-eat
SNP: Single nucleotide polymorphism
ST: Sequence type
WBC: White blood cell
WGS: Whole genome sequencing
